# Performance of deep learning models in predicting the nugent score to diagnose bacterial vaginosis

**DOI:** 10.1128/spectrum.02344-24

**Published:** 2024-11-19

**Authors:** Naoki Watanabe, Tomohisa Watari, Kenji Akamatsu, Isao Miyatsuka, Yoshihito Otsuka

**Affiliations:** 1 Department of Clinical Laboratory, Kameda Medical Center, Kamogawa, Chiba, Japan; 2 CarbGeM Inc., Tokyo, Japan; Icahn School of Medicine at Mount Sinai, New York, New York, USA

**Keywords:** artificial Intelligence, bacterial vaginosis, deep learning, diagnosis, Gram stain, machine learning, Nugent score

## Abstract

The Nugent score is a commonly used diagnostic tool for bacterial vaginosis. However, its accuracy depends on the skills of laboratory technicians. This study aimed to evaluate the performance of deep learning models in predicting the Nugent score to improve diagnostic consistency and accuracy. In total, 1,510 vaginal smear images collected from a hospital in Japan between 2021 and 2023 were assessed. Each image was annotated by laboratory technicians into one of four categories based on the Nugent score—normal vaginal flora, no vaginal flora, altered vaginal flora, or bacterial vaginosis. Deep learning models were developed to predict these categories, and their performance was compared to that of technician annotations. The deep learning models demonstrated 84% accuracy at 400× magnification and 89% at 1,000× magnification. The 1,000× model was further optimized and tested on an independent set of 106 images. After optimization, the advanced model achieved 94% accuracy, outperforming the average 92% accuracy of the technicians. The agreement between the advanced model predictions and technicians was 92% for normal vaginal flora, 100% for no vaginal flora, 91% for altered vaginal flora, and 100% for bacterial vaginosis. Overall, our findings suggest that deep learning models have the potential to diagnose bacterial vaginosis with an accuracy comparable to that of laboratory technicians.

**IMPORTANCE:**

Bacterial vaginosis is a global health issue affecting women, causing symptoms such as abnormal vaginal discharge and discomfort. The Nugent score is a standard method for diagnosing bacterial vaginosis and is based on the manual interpretation of Gram-stained vaginal smears. However, this method relies on the skill and experience of trained professionals, leading to variability in results and poses significant challenges for settings with limited access to experienced technicians. The results of this study indicate that deep learning models can predict the Nugent score with high accuracy, offering the potential to standardize the diagnosis of bacterial vaginosis. By reducing observer variability, these models can facilitate reliable diagnoses, even in settings where experienced personnel are scarce. Although validation is needed on a larger scale, our results suggest that deep learning models may represent a new approach for diagnosing bacterial vaginosis.

## INTRODUCTION

Bacterial vaginosis (BV) is a prevalent vaginal condition characterized by a shift from the normal *Lactobacillus* species to *Gardnerella vaginalis* and other BV-associated bacteria (1). It affects 23–29% of women worldwide, with regional variations (2). BV is associated with the risk of sexually transmitted infections, including *Chlamydia trachomatis*, *Trichomonas vaginalis* (3), *Mycoplasma genitalium* (4), human papillomavirus (5), and herpes simplex virus type 2 (6). BV is also associated with preterm birth in pregnant women (7) and neonatal complications (8).

BV is typically diagnosed using Amsel’s diagnostic criteria (9) and the Nugent score, which is determined by vaginal smear Gram staining (10). The Amsel criteria evaluate clinical symptoms and signs (9), whereas the Nugent score, ranging from 0 to 10, reflects the bacterial patterns in vaginal specimens (10). The Nugent score is valued for its low cost, quick turnaround time, and minimal equipment requirements. However, its accuracy varies depending on the skill and experience of the clinician.

Recent advances in deep learning, particularly convolutional neural network (CNN) (11), have shown promise for pattern recognition in images and speech, with potential applications in medical image classification. In infectious disease research, CNNs have been used for the automated interpretation of blood culture Gram staining (12) and BV classification (13). Wang et al. developed a CNN model to classify Nugent scores into three categories using high-magnification microscopic images, achieving 82% sensitivity and 97% specificity (13). Despite the potential of CNNs for diagnosing BV, improving their accuracy and automation capabilities remains challenging.

In this study, we developed a CNN-based bacterial vaginosis prediction model to classify vaginal smear images into four groups according to the Nugent score (BV model). The Nugent score traditionally uses three categories, with scores of 4–6 interpreted as altered vaginal flora. However, a score of 4 may indicate the absence of vaginal flora rather than their alteration. Considering the different microscopic patterns of altered and absent vaginal flora, we developed the BV model to accurately differentiate between these conditions. Furthermore, the BV models were evaluated using both 400× (low magnification) and 1,000× (high magnification) images. Images captured at low magnification do not require oil immersion, thus simplifying the process and facilitating automation.

## RESULTS

### Prediction performance of the BV model at low and high magnifications

In the initial evaluation, BV models were developed using 1,510 images, and their prediction performance was subsequently assessed. The images were classified according to Nugent scores as follows: In total, 320 images were classified as belonging to the normal vaginal flora category, 300 to the no vaginal flora category, 190 to the altered vaginal flora category, and 700 to the BV category. Table 1 shows the agreement between the predicted classifications of the BV model and true label groups. The high-magnification model accurately predicted 277 of the 310 samples based on the Nugent score, whereas the low-magnification model identified the correct category in 260 of the 310 samples.

**TABLE 1:** Prediction groups of the low-magnification and high-magnification models for true labels^*a*^

True label	No. of samples, low-magnification model				No. of samples, high-magnification model			
	Normal	No flora	Altered	BV	Normal	No flora	Altered	BV
Normal (*n* = 70)	70	0	0	0	56	0	14	0
No flora (*n* = 60)	0	60	0	0	0	59	0	1
Altered (*n* = 40)	8	0	12	20	0	0	38	2
BV (*n* = 140)	0	10	12	118	0	1	15	124

^*a*^ Normal, normal vaginal flora; No flora, no vaginal flora; Altered, altered vaginal flora; BV, bacterial vaginosis.

In the four-group classification, the high-magnification model outperformed the low-magnification model in terms of agreement rates across all categories (Table 2). The lowest agreement rate was observed for altered vaginal flora, with the high-magnification model at 57% and the low-magnification model at 50%. The high-magnification model achieved an accuracy of 89%, which surpassed the 84% accuracy of the low-magnification model. Of the 310 samples, 130 were classified as non-BV and the remaining 180 were classified as BV. In the two-group classification, the low-magnification model achieved 94% accuracy (292/310), which was slightly lower than the 95% accuracy of the high-magnification model (294/310). For the BV group, the low-magnification model demonstrated a 100% agreement rate, superior to that of the high-magnification model (92%). In the non-BV group, the low-magnification model exhibited a lower agreement rate (88%) compared to that of the high-magnification model (99%).

**TABLE 2:** Comparison of prediction performance between the low-magnification and high-magnification models^*a*^

Evaluation index	Performance of CNN model	
	Low-magnification model	High-magnification model
Agreement rates in four-group classifications (%)		
Normal vaginal flora (*n* = 70)	90	100
No vaginal flora (*n* = 60)	86	98
Altered vaginal flora (*n* = 40)	50	57
Bacterial vaginosis (*n* = 140)	86	98
Agreement rates in two-group classifications (%)		
Bacterial vaginosis (*n* = 180)	100	92
No bacterial vaginosis (*n* = 130)	88	99
Accuracy, (%)		
Four-group classifications	84	89
Two-group classifications	94	95

^*a*^ The agreement rate is defined as the percentage of results from the CNN model that matches the true label.

### Development and provisional performance of the advanced BV model

The high-magnification model, which initially exhibited superior accuracy, was further improved through additional learning. For this purpose, the advanced BV model was developed using a total of 1,940 images, including 430 new images. The revised image distribution included 450 images of normal vaginal flora, 490 images of no vaginal flora, 300 images of altered vaginal flora, and 700 images of bacterial vaginosis. In the interim evaluation, the advanced BV model achieved an accuracy rate of 92% in the four-group classification, representing a 3% improvement over an earlier version of the model.

### Comparison of the performances of the advanced BV model and laboratory technicians in predicting BV in the four-group classification

The performance of the advanced BV model was compared with that of laboratory technicians using an independent test set of 106 images. Table 3 shows the agreement between the predicted classifications of the advanced BV model and true label groups. For four-group classification, the advanced BV model achieved an accuracy of 94% (Table 4). The accuracies observed for the two laboratory technicians were 87% and 96%, respectively, and the collective average accuracy for the laboratory technicians was 92%. The lowest prediction accuracy was achieved for altered vaginal flora, whereas the advanced BV model showed a 91% agreement rate. The advanced BV model misclassified four images of altered vaginal flora and one image of bacterial vaginosis as normal vaginal flora.

**TABLE 3:** Predicted groups of the advanced BV model and technicians for independent test sets^*a*^

Model or technician	Group	True label			
		Normal (*n* = 61)	No flora (*n* = 10)	Altered (*n* = 14)	BV (*n* = 21)
Advanced BV model	Normal	61	0	4	1
	No flora	0	10	0	0
	Altered	0	0	10	1
	BV	0	0	0	19
Technician 1	Normal	61	0	2	5
	No flora	0	10	0	0
	Altered	0	0	11	6
	BV	0	0	1	10
Technician 2	Normal	58	0	0	0
	No flora	0	10	0	0
	Altered	3	0	13	0
	BV	0	0	1	21

^*a*^ Normal, normal vaginal flora; No flora, no vaginal flora; Altered, altered vaginal flora; BV, bacterial vaginosis.

**TABLE 4:** Comparison of prediction performance between the advanced BV model and technicians^*a*^

Group and evaluation index	Advanced BV model	Technician average	Technician 1	Technician 2
Agreement rates in four groups (%)				
Normal vaginal flora (*n* = 61)	92	95	90	100
No vaginal flora (*n* = 10)	100	100	100	100
Altered vaginal flora (*n* = 14)	91	73	65	81
Bacterial vaginosis (*n* = 21)	100	93	91	95
Agreement rates in two groups (%)				
Bacterial vaginosis (*n* = 35)	100	96	100	92
No bacterial vaginosis (*n* = 71)	93	96	91	100
Accuracy				
Four group (%)	94	92	87	96
Two group (%, 95% CI)	95 (89–99)	95 (NA)	93 (87–97)	97 (92–99)
Sensitivity (%, 95% CI)	86 (70–95)	90 (NA)	80 (63–92)	100 (86–100)
Specificity (%, 95% CI)	100 (93–100)	98 (NA)	100 (93–100)	96 (88–99)

^*a*^ NA, not applicable; Technician, laboratory technician.

### Comparison of the performances of the advanced BV model and laboratory technicians in predicting BV in the two-group classification

In the two-group classification, both the advanced BV model and technicians demonstrated sensitivities greater than 80%, specificities greater than 96%, and accuracies greater than 93%. The sensitivity of the advanced BV model was 86% (95% confidence interval [CI]: 70–95%), which was 4% lower than the average sensitivity of 90% achieved by the technicians. Conversely, the specificity of the advanced BV model was 100% (95% CI: 93–100%), which was 2% higher than that of the technicians. The overall accuracy of the advanced BV model was 95% (95% CI: 89–99%), which was comparable to the average accuracy reported by the technicians. The advanced BV model achieved an overall agreement rate of 92% with both laboratory technician 1 and laboratory technician 2. The kappa coefficient indicated an almost perfect agreement of 0.81 (range: 0.68–0.94) between the advanced BV model and technician 1, and an almost perfect agreement of 0.83 (range: 0.71–0.94) with technician 2. The inter-technician agreement rate was 91%, with a kappa coefficient of 0.78 (range: 0.65–0.91), indicating substantial agreement between technician 1 and technician 2.

### Diagnostic performance of the advanced BV model using a confidence score cutoff

In the four-group classification, the median confidence score was 0.79 (interquartile range, 0.77–0.80) for correctly predicted images and 0.68 (0.61–0.74) for incorrectly predicted images. A receiver operating characteristic curve was constructed using confidence scores as the predictor and correct predictions of the BV group as the outcome. The area under the curve for the four-group classification was 0.852 (95% CI, 0.718–0.985), with a confidence score cutoff of 0.62 required to achieve a sensitivity of at least 95%. Upon applying this cutoff value to an independent test set of 106 images, seven images (7%) were identified with scores below the threshold. Of the remaining 99 images, the advanced BV model correctly identified the BV group for 95 images (96%) in the four-group classification. This represented a 2% improvement in accuracy compared to the results obtained without this cutoff, yielding an accuracy rate of 94%. Similarly, in the two-group classification, the accuracy with the 0.62 cutoff (97%) outperformed the accuracy obtained without the cutoff (95%).

## DISCUSSION

We developed a CNN model to predict Nugent scores, and this model achieved 94% accuracy across a four-group classification. This result surpassed the performance reported by Wang et al. (13), who achieved 80% accuracy for three Nugent score groups in a test set created from images captured taken at a single facility. Our CNN model differs from that proposed by Wang et al. with respect to the underlying base model, which includes an additional Nugent score group. Our approach used ConvNeXt (14), which differed from the EfficientNet (15) used by Wang et al. (13). Further, their model categorized scores into three groups, whereas our study expanded these to four groups. These changes likely contributed to the improved model accuracy.

Our model effectively matched the laboratory technicians with an accuracy of 95%, sensitivity of 86%, and specificity of 100% in the two-group classification. Wang et al. reported a sensitivity of 89% and a specificity of 85% (13). As observed in the model developed by Wang et al., our model demonstrated a sensitivity of less than 90%. The primary misclassification occurred in samples with altered vaginal flora, which were incorrectly identified as normal flora. Furthermore, the accuracy of identifying altered vaginal flora also presented a challenge for human technicians, as evidenced by the low average agreement rate of 73%. Therefore, the accuracy of the CNN model must be improved, particularly for samples with altered vaginal flora.

Low-magnification images offer advantages, primarily because of their ease of acquisition and rapid collection process. Additionally, they are well-suited for implementation on automated microscopy platforms. For example, Smith et al. used an automated microscopy platform for collecting Gram-stained images at 400× magnification to develop a CNN model (12). In our study, the low-magnification model demonstrated an accuracy of 84% for the four-group classification, exhibiting a 5% discrepancy from the accuracy of the high-magnification model (89%). This discrepancy is less than the 9% accuracy gap observed between technician 1 (87%) and technician 2 (96%). Considering the inter-technician variability observed in the Nugent score classification, a 5% discrepancy can be deemed clinically acceptable. Although the low-magnification model exhibits a slight decline in accuracy, its benefits in terms of image acquisition efficiency make it a viable option. However, further validation is essential to evaluate the clinical implications of this reduced accuracy.

BV is a common condition in women, typically diagnosed using conventional methods and nucleic acid amplification tests (16–18). Conventional methods include the Nugent score (10), Amsel’s diagnostic criteria (9), OSOM BV Blue assay (19, 20), and FemExam card (21). Nucleic acid amplification tests, such as the BD Max vaginal panel (22) and Hologic Aptima BV (23), are also used for diagnosis. The Nugent score demonstrates substantial inter-observer agreement with kappa coefficients ranging from 0.70 to 0.77 (24) and inter-center agreement ranging from 0.60 to 0.72 (25). However, interpretation of the Nugent score requires expertise, which affects its reproducibility. The BV model exhibits high concordance rates with technician interpretations (kappa coefficients of 0.81–0.83) and provides results that are independent of technician skill and subjectivity. Implementation of the BV model in a clinical setting could thus facilitate objective and reproducible interpretation of vaginal smear Gram staining.

This study has limitations, particularly in terms of generalizability and sample size. The evaluation was confined to a single institution, which may have limited the broader applicability of the results. Factors such as sample diversity, variations in image hue, and technician skills, which may vary among institutions, could affect the model accuracy. Furthermore, the BV model was developed using a relatively modest data set of less than 2,000 samples, which may have resulted in undertraining and affected predictive ability. Despite these limitations, our BV model demonstrated sensitivity, specificity, and accuracy comparable to those of technicians. With an expanded data set, we anticipate significant improvements in the predictive performance of the model, further refining its effectiveness for BV diagnosis when tested on a broader range of samples and settings.

The BV model demonstrates high accuracy in predicting Nugent scores and thus has the potential to serve as a diagnostic support tool for BV. Furthermore, the incorporation of a confidence score threshold to prompt additional human review for inconclusive outcomes can improve prediction precision while reducing the workload on experts. Our analysis revealed that 7% of the images exhibited a score below the established cutoff. This suggests that employing this threshold could restrict human involvement to 7% of cases, reducing the microscopy workload to approximately one-fifteenth of the total. Furthermore, the application of a cutoff improved the accuracy of the advanced BV model to 96%. Our findings underscore the potential for CNN models to play a pivotal role in the automated classification of BV scores. Currently, limited data are available on the use of CNN models for BV prediction, and further validation using additional specimens and clinical settings is necessary to confirm their efficacy.

## MATERIALS AND METHODS

**Fig 1:**
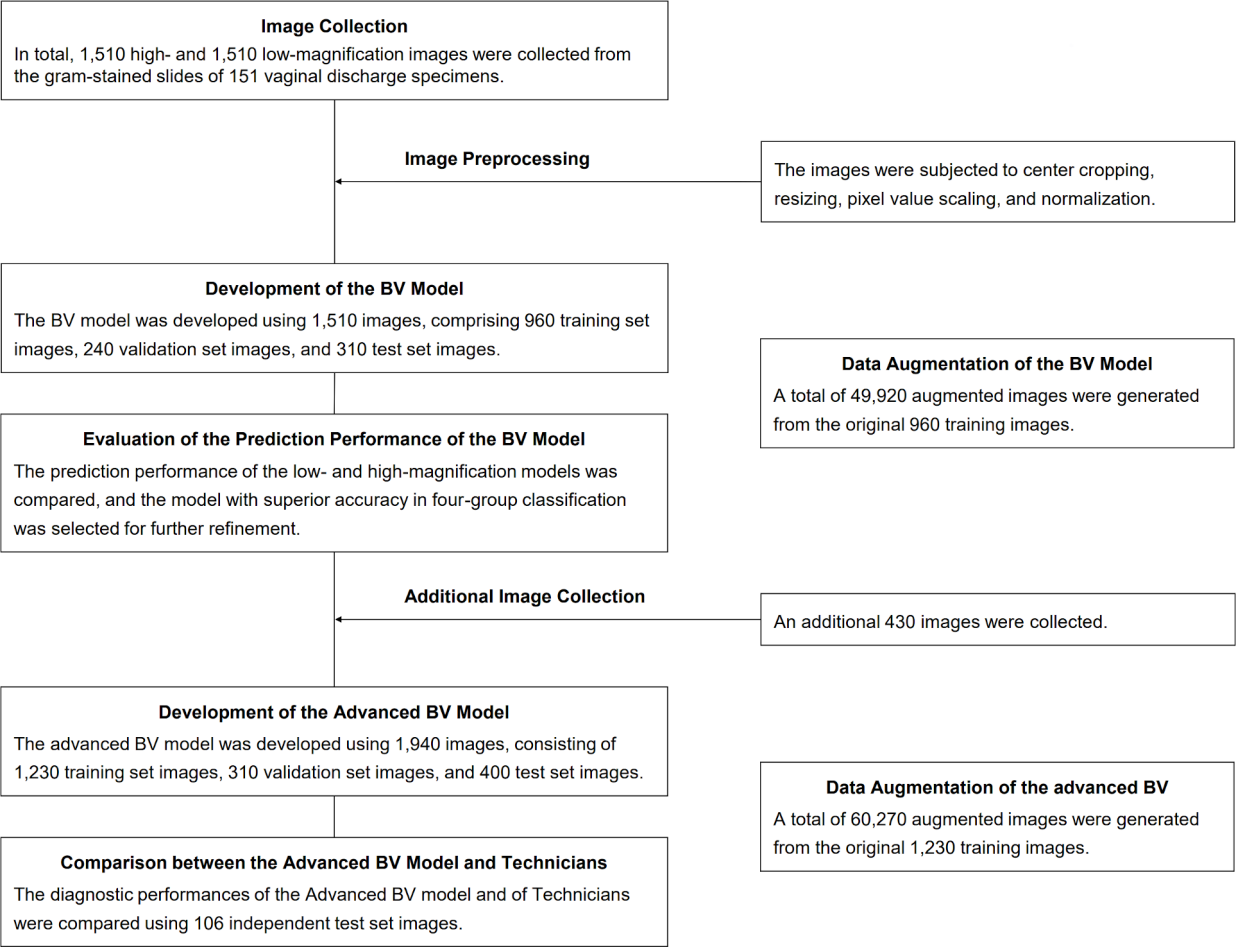
Flowchart of the development and evaluation of a CNN model for predicting bacterial vaginosis

This study was conducted at the Kameda Medical Center in Japan from November 2021 to February 2024. Figure 1 shows the flowchart of this study. After data collection and preprocessing, two magnification versions of the CNN model were developed for comparative evaluation. The more effective model of these was subsequently selected, improved, and subjected to final evaluation. Ethical approval was obtained from the Kameda Medical Center Ethics Committee (approval number 22-128). The requirement for written informed consent from the participants was waived by the Research Ethics Committee because of the exclusive use of anonymized data in this study.

### Data collection

From November 2021 to May 2023, a total of 151 vaginal discharge specimens were collected and prepared as Gram-stained slides. For each slide, 10 images were captured at both low and high magnifications, resulting in a total of 1,510 images per magnification. Gram staining was performed using Neo-B & M Wako crystal violet solution, iodine solution, decolorizing solution, and Pfeifel solution (FUJIFILM Wako Chemicals, Osaka, Japan). The images were obtained through manual capture using a Nikon Eclipse Ci-S microscope equipped with a DS-Fi3 digital camera. The images, focused on regions where bacteria or cells were visible, were captured at low and high magnifications, each with a resolution of 2,880 × 2,048 pixels.

**Fig 2:**
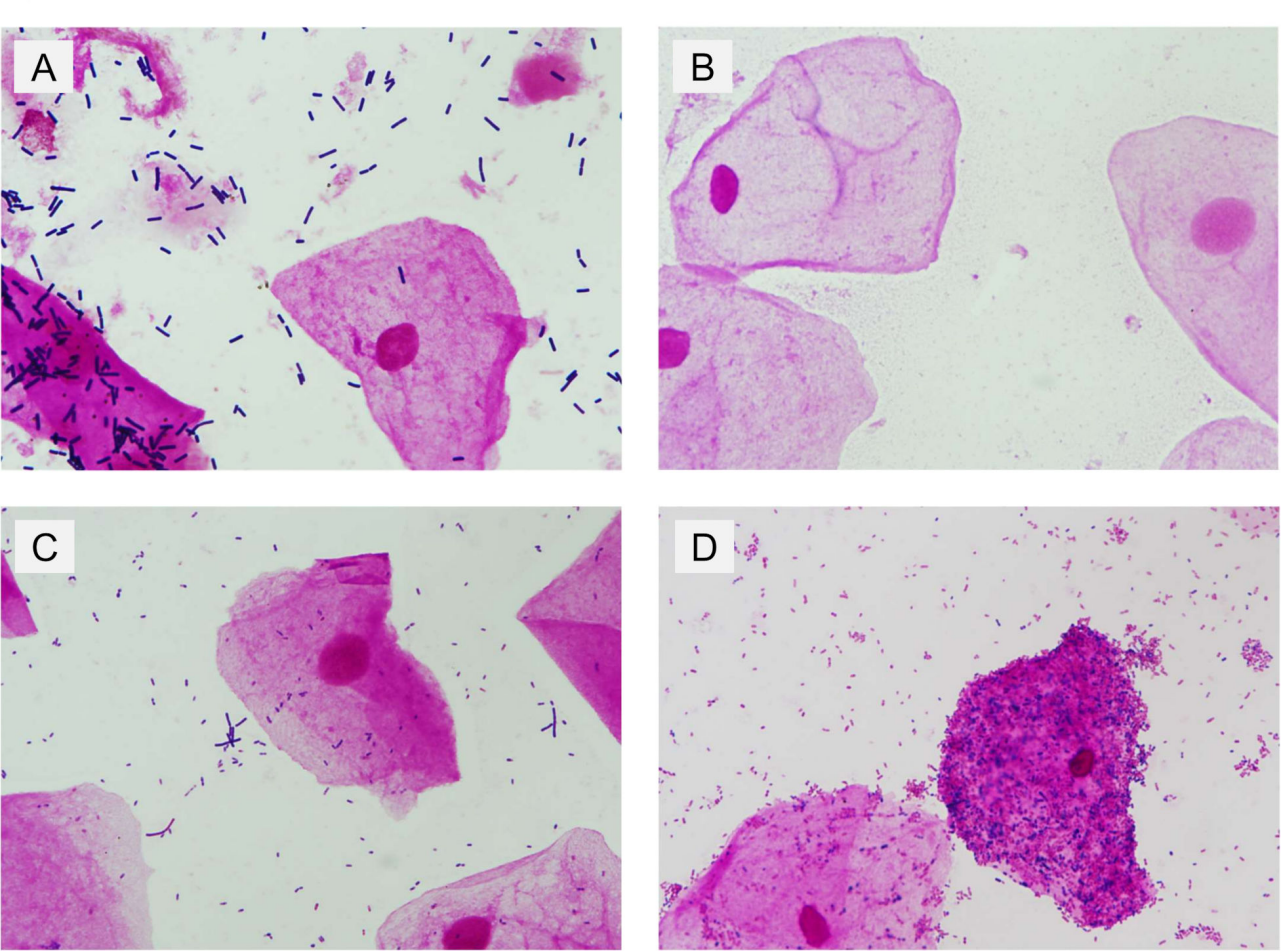
Microscopic images of vaginal discharge specimens in each Nugent score category. Description: Representative high-magnification images of vaginal discharge specimens, each categorized by the Nugent score. The images are labeled as follows: Image A, Nugent score of 0–3 (normal vaginal flora); Image B, score 4 (no vaginal flora); Image C, scores 5 and 6 (altered vaginal flora); and Image D, scores 7–10 (bacterial vaginosis).

Images were categorized into four groups according to the Nugent score: normal vaginal flora (score 0–3); no vaginal flora (score 4), altered vaginal flora (scores 5 and 6); or BV (scores 7–10). Figure 2 shows the representative slide images for each group. Nugent scores were assessed by two laboratory technicians, including at least one certified clinical microbiology specialist. In case of disagreement, a third technician was consulted for the final decision. To prevent the assignment of multiple images from the same slide to disparate data sets, these images were divided into training, validation, and test sets, with each slide serving as the smallest unit. Consequently, the images were randomly assigned to the training, validation, and test sets, with 960, 240, and 310 images, respectively, assigned to each set.

### Image preprocessing and data augmentation

We applied four preprocessing steps to the collected images: center cropping, resizing, scaling pixel values, and normalization of pixel values. Microscopic images were cropped from their original size of 2,880 × 2,048 pixels to a central area of 2,048 × 2,048 pixels. The cropped images were resized to 1,024 × 1,024 pixels. The pixel values were scaled from (0, 255) to (0, 1) and normalized to RGB means of (0.485, 0.456, and 0.406) and RGB standard deviations of (0.229, 0.224, and 0.225).

To increase the size of the training data set and improve model performance, data augmentation techniques were applied. These techniques included random rotation, random cropping, random horizontal and vertical flipping, random affine transformations, and color jittering. Random rotation and cropping involved arbitrary rotations and adjustments of image dimensions. Random horizontal and vertical flipping altered images by flipping them left/right and up/down, respectively. Random affine transformations and color jittering variably adjusted the affine parameters of brightness, contrast, saturation, and hue. The BV model was subjected to 52 training iterations, with data augmentation applied at each iteration. Consequently, a total of 49,920 augmented images were generated from the original 960 training images and employed for model training.

### Development of a CNN-based model for Nugent score prediction

Neural networks are mathematical models that emulate the functions of nerve cells in the human brain. Specifically, in image classification, these networks learn to recognize image content by iteratively processing the training data, thereby updating the connections between neurons. Among the various types of neural networks, CNNs are tailored to process image data. In our study, we used a model based on ConvNeXt, a variant of a CNN known for its state-of-the-art performance in image classification, including its high accuracy and scalability (14). We used a linear activation function in the final layer of the BV model to compute the probabilities representing the likelihood of each Nugent score group. This step was essential for effectively predicting Nugent scores based on the analyzed images.

### Evaluation of the prediction performance of the BV model

The BV model outputted confidence scores for each category, and the group with the highest confidence score was selected as the predicted group. The predictive performance of the BV model was evaluated for both the four- and two-group classifications derived from the BV categories. For the two-group classification, the four Nugent scores were divided into two categories: BV and non-BV, with normal and no vaginal flora were categorized as non-BV whereas altered vaginal flora and bacterial vaginosis were categorized as BV. We used agreement rate and accuracy as the evaluation metrics. The agreement rate measures the consistency between the BV model predictions and true labels and is expressed as a percentage. Accuracy is the proportion of correct predictions made by the BV model compared with the actual labels over the entire data set. For the two-group classifications, sensitivity and specificity were calculated as follows: sensitivity was the ratio of correctly predicted BV cases to the total number of actual BV cases; and specificity was the ratio of correctly predicted non-BV cases to the total number of actual non-BV cases.

### Development of an advanced BV model

Among the models developed using low-magnification and high-magnification images, the model with superior accuracy in the four-group classification was selected for further refinement. This further development process included the integration of 430 additional images, collected between August and October 2023. These were used with the same methodology as in the initial phase of model development. This brought the total number of images used in the development of the advanced BV model to 1,940. The images were randomly assigned to the training, validation, and test sets, with 1,230, 310, and 400 images, respectively, assigned to each set. The advanced BV model was subjected to 49 training iterations, with RandAugment (26) applied at each iteration. Consequently, a total of 60,270 augmented images were generated from the original 1,230 training images and employed for model training. The performance of this advanced BV model was assessed on an interim basis using the same test set of 310 images used in the initial evaluation.

### Accuracy comparison between the advanced BV model and human assessment in BV diagnosis

An independent test set was used to compare the accuracy of the advanced BV model with that of laboratory technicians. For this evaluation, one image was obtained from each of the 106 vaginal discharge specimens collected in December 2023. Of the specimens, 61 (58%) had normal vaginal flora, 10 (9%) had no vaginal flora, 14 (13%) had altered vaginal flora, and 21 (20%) had BV. Consequently, in the two-group classification, 71 specimens (67%) were identified as non-BV, while 35 specimens (33%) were classified as BV. The data were employed to evaluate the agreement rate, accuracy, and kappa coefficients between the advanced BV model and laboratory technicians. The agreement rate was evaluated by calculating the kappa coefficient for the two-group classification.

The accuracy of the advanced BV model was evaluated by applying a confidence score cutoff. A ROC curve was generated using confidence scores as predictors and correct BV group predictions as outcomes, with the cutoff defined as the confidence score required to achieve at least 95% sensitivity. Subsequently, this cutoff was applied to an independent test set consisting of 106 images, allowing for calculating the proportion of cases falling below the cutoff and the accuracy of the advanced BV model in the remaining images. Statistical analyses were conducted using EZR version 1.64 (27).

## Data Availability

The data from the independent test set and the confidence scores of the advanced BV model have been deposited in the figshare (28).
